# Methylation of MGMT promoter does not predict response to temozolomide in patients with glioblastoma in Donostia Hospital

**DOI:** 10.1038/s41598-020-75477-9

**Published:** 2020-10-28

**Authors:** Larraitz Egaña, Jaione Auzmendi-Iriarte, Joaquin Andermatten, Jorge Villanua, Irune Ruiz, Alejandro Elua-Pinin, Paula Aldaz, Arrate Querejeta, Cristina Sarasqueta, Felix Zubia, Ander Matheu, Nicolas Samprón

**Affiliations:** 1 Donostia University Hospital, Paseo Dr. Beguiristain, 20014 San Sebastian, Spain; 2 Biodonostia Health Research Institute, San Sebastian, Spain; 3 Osatek Institute of Radiology, San Sebastian, Spain; 4 University of the Basque Country, Bilbao, Spain; 5 Ikerbasque, Basque Foundation for Science, Bilbao, Spain; 6 CIBERfes, Madrid, Spain

**Keywords:** Molecular medicine, Oncology

## Abstract

O^6^-methylguanine-DNA methyltransferase (MGMT) promoter methylation status has been considered a prognostic factor in newly diagnosed glioblastoma (GBM). In this study, we evaluated the prognostic and predictive value of MGMT promoter methylation in patients with glioblastoma in Donostia Hospital. Surprisingly, methylation of MGMT promoter did not predict response to temozolomide in patients with glioblastoma in Donostia Hospital. Specifically, overall survival (OS) and progression-free survival (PFS) did not differ significantly by MGMT methylation status in our cohort. In contrast, both were longer in patients who received treatment, received more TMZ cycles, had a better general status and perform at least a partial resection. No association was detected between methylation of MGMT promoter and molecular markers such as ATRX, IDH, p53 and Ki67. These results indicate that MGMT methylation did not influence in patient survival in our cohort.

## Introduction

Glioblastoma (GBM) is the most common malignant tumour of the central nervous system in adulthood. The median age at diagnosis is 64 years old. Though it accounts for only about 1.4% of all cancers diagnosed, its incidence is rising, especially in elderly people, and it is highly aggressive^1^: it is associated with high levels of mortality, morbidity and disability, and patients have an expected survival of just 16 months^2^. Since 2005, the first-line treatment has consisted of surgery (seeking to achieve the widest possible resection), followed by adjuvant therapy consisting of radiotherapy (RT) and temozolomide (TMZ). A 5-year survival rate of as high as 8% has been reported with this treatment^3,4^.

It is known that TMZ inhibits the replication of DNA in the tumour through the alkylation of guanine at the *O*^6^ position. The enzyme *O*-6-methylguanine-DNA methyltransferase (MGMT) removes the alkyl groups from guanine at the *O*^6^ position, thereby counteracting the effect of TMZ^5^. Methylation of the MGMT gene promoter silences the expression of the gene, leading to a reduction in levels of the MGMT enzyme. Various studies have found an association between MGMT promoter methylation and longer survival in patients diagnosed with GBM treated with TMZ, both in the overall population and in patients over 64 years of age^6–10^.

In this context, we sought to assess whether methylation of the MGMT promoter was a predictor of response to TMZ in patients diagnosed with GBM in our hospital; additionally, we wanted to determine whether its predictive value is independent of age, functional status and relevant molecular markers.

## Material and methods

### Patient information

An observational retrospective study was conducted including 334 patients over 16 years of age, diagnosed with and treated for GBM and high-grade glioma between 15 January 2003 and 31 July 2017 at Donostia University Hospital. We excluded cases of GBM initially diagnosed as low-grade glioma that evolved to GBM over time. 18 of 334 patients were anaplastic astrocytoma, the rest were glioblastoma or high-grade glioma in the biopsy but with glioblastoma radiological features. The cases were tumor sample were tested for MGMT methylation status were glioblastomas. The study was approved by the ethic committee for clinical research of Euskadi and all patients gave written informed consent before inclusion. In case of participants under the age of 18 years informed consent was obtained from a parent and/or legal guardian. All methods were performed in accordance with the relevant guidelines and regulations.

Treatment was provided following the Stupp protocol^3^. The RT consisted of a 60-Gy dose given in 30 fractions; during this time, oral TMZ was administered daily at a dose of 75 mg/m^2^/day. The treatment regimen for TMZ monotherapy was six cycles of TMZ and each cycle involved a dose of 150–200 mg/m^2^/day for 5 days every 28 days.

Patient follow-up was based on physical examination where Karnofsky performance status (used as an indicator of general status) ^11^ and *Mini-Mental State Examination* (MMSE)^12^ were performed and magnetic resonance imaging. The first imaging follow-up after RT was carried out 6 weeks after the end of the RT, and subsequently, imaging was performed every 3 months for the first 2 years, then every 6 months from year 2 to year 5, and once a year thereafter.

### MGMT methylation

Bisulfite modification of DNA allows the identification of CpG sites methylated. DNA modification was developed using genomic DNA (1 μg) from primary tumors, which was denatured by NaOH (0.2 M) for 10 min at 37 °C and then modified by hydroquinone and sodium bisulfite treatment for 17 h under a mineral oil layer at 50 °C. Modified DNA was purified using the Wizard DNA Clean-Up system (Promega). Modification was completed by NaOH (0.3 M) treatment for 5 min at room temperature, followed by precipitation with glycogen, 10 M ammonium acetate and ethanol precipitation.

Bisulfite modified primary tumor DNAs were used to analyze the *MGMT* methylation status by methylation-specific PCR (MSP) using specific primers to detect methylated or unmethylated modified DNA specifically described^13^, following protocol described in Ibanez Caceres et al.^14^. MSP amplification of tumor DNA was performed for the unmethylated reaction: 35 cycles at 95 ^0^C denaturing, 60 ^0^C annealing while methylated reaction: 37 cycles at 95 ^0^C denaturing, 68 ^0^C annealing both reactions had a 72 ^0^C extension with a final extension step of 5 min. Each set of DNAs modified and PCR amplified, includes (i) human lymphocyte DNA from healthy donors as negative control (−), (ii) human lymphocyte DNA from healthy donors in vitro methylated with *Sss* I methylase, according to the manufacturers instructions (New England Biolabs), used as a positive control ( +) and (iii) water with no DNA template as a control for contamination. Samples were run then on a 6% non-denaturing acrylamide gel and the presence or absence of a PCR product analyzed.

### Pirosequencing

Bisulfite-converted DNA was used to amplify the MGMT promoter with the primers provided by the Pyromark CpG MGMT kit. A PCR was set up with reagents and conditions given by the PyroMark kit. PCR products were attached to Streptavidina Sepharose perls and the template strands were purified in the Pyromark Q24 Vaccum Workstation. The purified templates were incubated for 20 min with the sequencing primer provided by the kit and run in the instrument *Qiagen Pyromark Q24 System* with the reactives of Pyromark Gold Q24 Reagents. All the steps were followed according user manual. Sequencing conditions and analysis of results were performed with the Pyromark Q24 sofware 2.0.8. We have established 3 subgroups divided as unmethylated (0–8%), methylated (13–100%) and a pattern indicative of low/moderate methylation (9–12%) following a previously described classification^15^.

### Immunohistochemistry of glioblastoma sections

IHC was performed following standard procedures. Briefly, tumor tissues were extracted, fixed in formalin, paraffin-embedded and subsequently sectioned in 5 µm coronal sections with a Leica rotary microtome. The following primary antibodies were used: Ki67 (Roche; Ref 790-4286, dilution 1:100), p53 (Roche; Ref 790-2912, predilution 1:20), IDH1 (vitro master diagnostica; Ref R132H antibody H09, predilution) and ATRX (Sigma-Aldrich; Ref HPA001906, 1:200). IHCs were analyzed by a senior pathologist of the Service and were performed following the manufacturer’s instructions on the *Roche Ventana Benchmark ULTRA System* with ethylenediaminetetraacetic acid (EDTA) pH 8.5 antigen retrieval. Sections were scanned with *Virtuoso* v.5.6.1 software (Ventana Medical Systems, Roche).

### Statistical analysis

To assess relationships between clinical variables and methylation status, Fisher’s and/or chi-square tests were performed for qualitative variables and Student’s t test or analysis of variance for quantitative variables. A multivariate analysis was then carried out using binary logistic regression. Overall survival (OS) was assessed from the date of the first surgery until patient death or the last follow-up or contact with the patient. Progression-free survival and recurrence was measured from the date of the first surgery until disease progression or death. The Kaplan–Meier method was used for calculating the median survival and probability of survival at 12 and 24 months. The association of clinical characteristics and survival with MGMT promoter methylation status was explored using the Log-Rank test. Subsequently, a Cox regression analysis was performed with the same variables. P values ≤ 0.05 were considered statistically significant. Statistical analysis was carried out using IBM SPSS version 23.

## Results

The mean age of patients at diagnosis was 60 years. At the time of the analysis, out of the 334 patients included, 290 died (86.82%) and 283 had recurrence (84.5%). Among the 290 who died, the primary cause of death was tumour progression in 259 cases (89.31%), just 30 patients (10.34%) dying without recurrence, due to complications, postoperatively or during or after RT and/or treatment with TMZ. Patient characteristics are summarised in Table 1.

**Table 1:** Patient characteristics.

	n	(%)
Age, years		
< 50	47	14.32
50–64	149	44.47
≥ 65	138	41.11
Sex		
Woman	139	41.8
Man	195	58.2
Karnofsky performance status, %		
> 80	45	13.4
70–80	114	34
60–70	74	22.1
< 60	75	22.4
Unknown	26	8.1
Mini-Mental State Examination		
≥ 25	59	17.6
≤ 24	14	4.2
Unknown	261	78.14
Extent of surgical excision		
Total/subtotal	149	44.8
Partial	84	25.4
Biopsy	100	29.9
Radiotherapy		
Yes	252	75.44
No	82	24.55
Concomitant temozolomide		
Yes	200	59.19
No	134	39.81
Adjuvant temozolomide (number of cycles)		
0	119	35.4
1–5	129	38.4
≥ 6	86	25.8
Hemispheric location of tumour		
Right	144	41.6
Left	170	49.1
Bilateral	32	9.2

**Figure 1:**
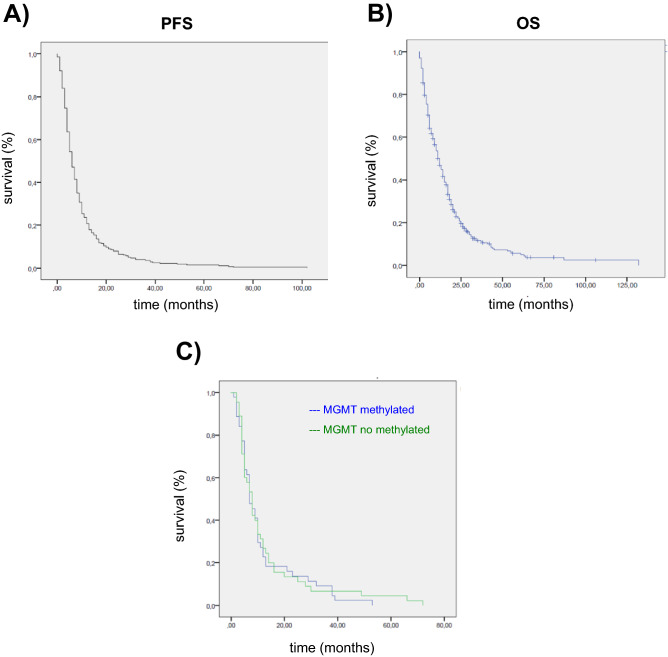
**(A)** Progression-free survival (PFS) and **(B)** overall survival (OS) of glioblastoma patients (n = 334) treated in Donostia University Hospital. **(C)** Patient survival curve based on MGMT promoter methylation status (n = 107).

The median progression-free survival (PFS) was 6 months (95% CI, 5.20–6.79). The 12- and 24-month PFS rates were 20.7% and 7.9%, respectively (Fig. 1A). The median OS was 12 months (95% CI 10.12–13.87) and the 12 and 24-month OS rates were 46.8% and 20.7%, respectively (Fig. 1B).

Out of the 334 patients included, measurements of MGMT levels were successfully carried out in 107 cases, a third of our patients (32.03%) and this subset was generally representative of the rest of the sample. Methylation was observed in 52 samples but not in the other 55 (Table 2). The majority of samples (up to 2016) were analysed using methylation-specific PCR (92 out of 107), whereas the latest 15 were performed using Pyrosequencing (Fig. Supplementary 1). The ratio of MGMT promoter methylation with each technique is 44 of 92 (47%) with MSP and 9 of 15 (60%) with Pyrosequencing. In our hospital, we have established three subgroups divided as unmethylated (0–8%), methylated (13–100%) and a pattern indicative of low/moderate methylation (9–12%) following a previously described classification^11^. Of note, 2 out of the 15 samples were assigned to the third category and were considered methylated in our analysis, and this may have contributed to the relatively higher rate of methylation observed using this technique (around 60% compared to 47.25% with methylation-specific PCR).

**Table 2:** Association between *O*-6-methylguanine-DNA methyltransferase methylation status and different variables.

	MGMT		
	Methylated, n (%)	Unmethylated, n (%)	p
**Age, years**			
< 50	8 (34.8)	15 (65.2)	
50–64	22 (45.8)	26 (54.2)	**0.043**
≥ 65	22 (61.1)	14 (38.9)	
**Sex**			
Female	30 (48.4)	32 (51.6)	0.5
Male	22 (48.9)	23 (51.1)	
**General status (KPS, %)**			
90–100	11 (52.4)	10 (47.6)	
70–80	17 (42.5)	23 (57.5)	0.71
60–70	12 (54.5)	10 (45.5)	
< 60	9 (52.9)	8 (47.1)	
**Type of surgery**			
Total/subtotal resection	29 (44.6)	36 (55.4)	
Partial resection	11 (45.8)	13 (54.2)	0.14
Biopsy	12 (66.7)	6 (33.3)	
**Treatment**			
Yes	45 (50)	45 (50)	0.34
No	7 (41.2)	10 (58.8)	
**Number of TMZ cycles**			
0	11 (44)	14 (56)	
1–5	22 (48.9)	23 (51.1)	0.5
≥ 6	19 (52.8)	17 (47.2)	
**Tumour localization**			
Right	29 (60.4)	19 (39.6)	
Left	23 (46.0)	26 (54.0)	0.165
Bilateral	2 (28.6)	5 (71.4)	

*KPS* Karnofsky Performance Status, *MGMT* *O*-6-methylguanine-DNA methyltransferase, *TMZ* temozolomide.

The mean age of patients with methylation was 60 years, while that of patients without MGMT methylation was 56 years. In our sample, the older the age the greater the probability of having methylated MGMT (61.1% among patients ≥ 65 years old, 45.8% among those between 50 and 64 years old, and 34.7% among under-50-year-olds, p < 0.043), although the differences were not found to be significant in the multivariate analysis (Tables 2 and 3). No significant differences in methylation status were found considering any of the other variables analysed such as sex, Karnofsky Index, Mini-Mental state examination, extent of surgical excision, the location of the tumour and therapeutic treatments (Tables 2 and 3), as well as in the survival of the patients (Fig. 1C).

**Table 3:** Association of *O*-6-methylguanine-DNA methyltransferase methylation status with age and type of surgery.

	Odds ratio	95% confidence interval	p
Age (≥ 65 years)	0.394	0.12–1.24	0.114
Age (46–64 years)	0.68	0.23–2.01	0.496
Type of surgical intervention (biopsy)	0.526	0.16–1.66	0.275

Multivariate analysis.

**Figure 2:**
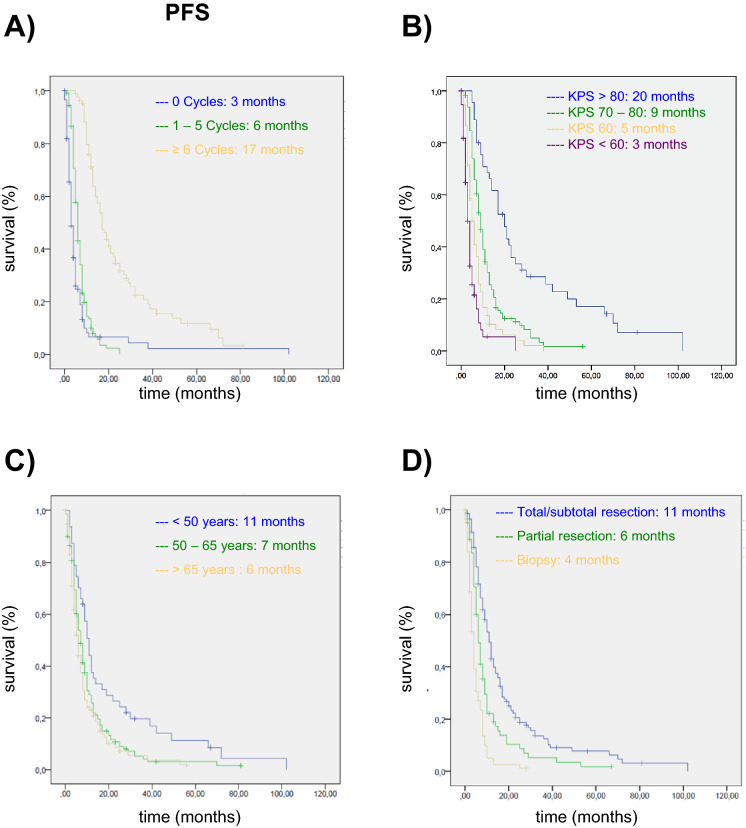
Progression-free survival (PFS) of glioblastoma patients treated in Donostia University Hospital stratified by **(A)** number of cycles of TMZ, **(B)** performance status stratified by Karnofsky performance status, **(C)** age, **(D)** type of surgery.

Analysing the association of PFS with the variables studied, The PFS was significantly longer in patients who received treatment after surgery, the larger the number of TMZ cycles they received (Fig. 2A), the better their general status (Fig. 2B), and the younger they were (Fig. 2C), as well as in those in whom it was possible to perform total or subtotal resection (Fig. 2D). Multivariate analysis was then carried out to determine whether the association found between PFS and each of these variables was independent of the others. Significant multiplicative interactions were found between age, number of cycles and type of surgery, and hence, the data were analysed separately. In this case, we observed a lower risk of recurrence among under-65-year-olds who received at least one cycle of TMZ. The reduction in risk was significantly greater among those who received 6 or more cycles (Table 4). Further, the risk of recurrence was significantly higher among those with a Karnofsky Performance Status score < 60%. In contrast, in patients aged ≥ 65 years old, neither number of cycles nor general status had a significant impact in the multivariate analysis (Table 4). In this group, the type of surgery did, with the risk of recurrence being lower in patients in whom it was possible to carry out at least subtotal resection. Notably, MGMT methylation status did not have any impact in either of the age groups studied (Table 4).

**Table 4:** Progression-free survival.

	Variables	Odds ratio	95% confidence interval	
< 65 years	MGMT	0.888	0.467–1.687	0.716
	1–5 cycles	0.35	0.126–0.972	0.044
	> 6 cycles	0.06	0.018–0.202	0.0001
	KPS 70–80%	1.98	0.925–4.247	0.078
	KPS 60–70%	2.33	0.747–7.285	0.145
	KPS < 60%	8.58	2.206–33.414	0.002
> 65 years	MGMT	0.360	0.097–1.339	0.127
	Total/subtotal resection	0.182	0.049–0.677	0.011
	> 6 cycles	0.200	0.018–2.155	0.184
	KPS < 60%	7.177	0.263–196.20	0.243

Multivariate analysis.

*KPS* Karnofsky performance status, *MGMT* *O*-6-methylguanine-DNA methyltransferase.

**Figure 3:**
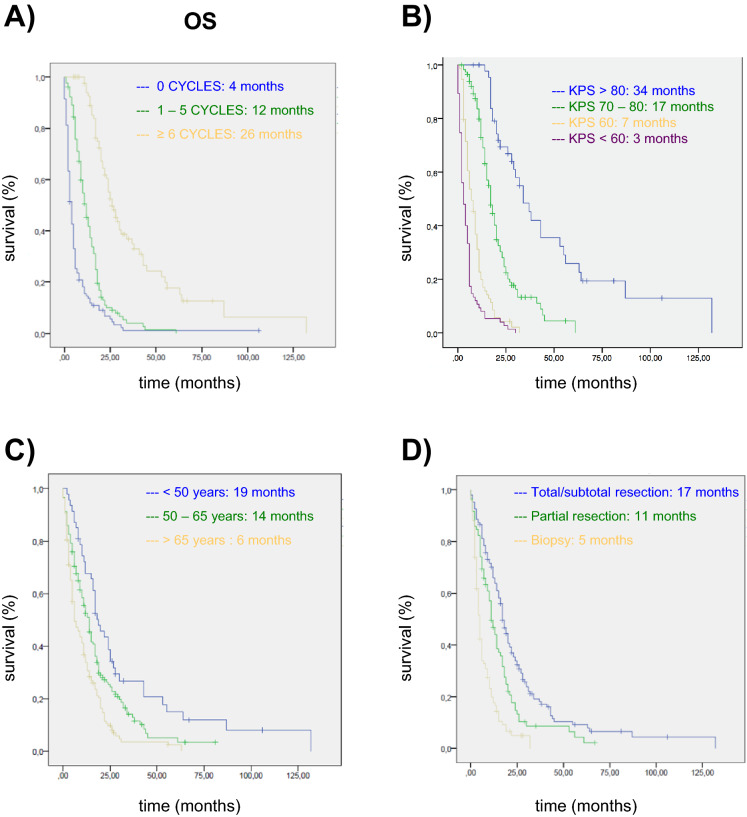
Overall survival (OS) of glioblastoma patients treated in Donostia University Hospital stratified by **(A)** number of cycles of TMZ, **(B)** performance status stratified by Karnofsky performance status, **(C)** age, **(D)** type of surgery.

Regarding OS, significant differences were found for the same variables as for PFS. Specifically, OS was longer in patients who received treatment (Fig. 3A), received more TMZ cycles (Fig. 3B), and had a better general status (Fig. 3C), as well as those in whom it was possible to perform at least a partial resection (Fig. 3D). In the multivariate analysis, we observed a significantly lower risk of death in patients in whom total or subtotal resection was performed, and in those who received at least 1 TMZ cycle (the reduction being greater the more cycles) (Table 5). On the other hand, the risk of death was higher the poorer the general status based on lower Karnofsky index. Once again, MGMT methylation status did not have any significant impact (Table 5).

**Table 5:** Overall survival.

Variables	Odds ratio	95% confidence interval	p
MGMT	0.861	0.528–1.406	0.551
Partial resection	0.641	0.310–1.328	0.231
Total/subtotal resection	0.329	0.140–0.771	**0.011**
1–5 cycles	0.484	0.245–0.956	**0.037**
≥ 6 cycles	0.163	0.071–0.337	**0.0001**
KPS 70–80%	2.602	1.276–5.307	**0.009**
KPS 60–70%	6.626	2.817–16.528	**0.0001**
KPS < 60%	6.961	2.783–17.411	** 0.0001**

Multivariate analysis.

*KPS* Karnofsky performance status, *MGMT* *O*-6-methylguanine-DNA methyltransferase.

**Figure 4:**
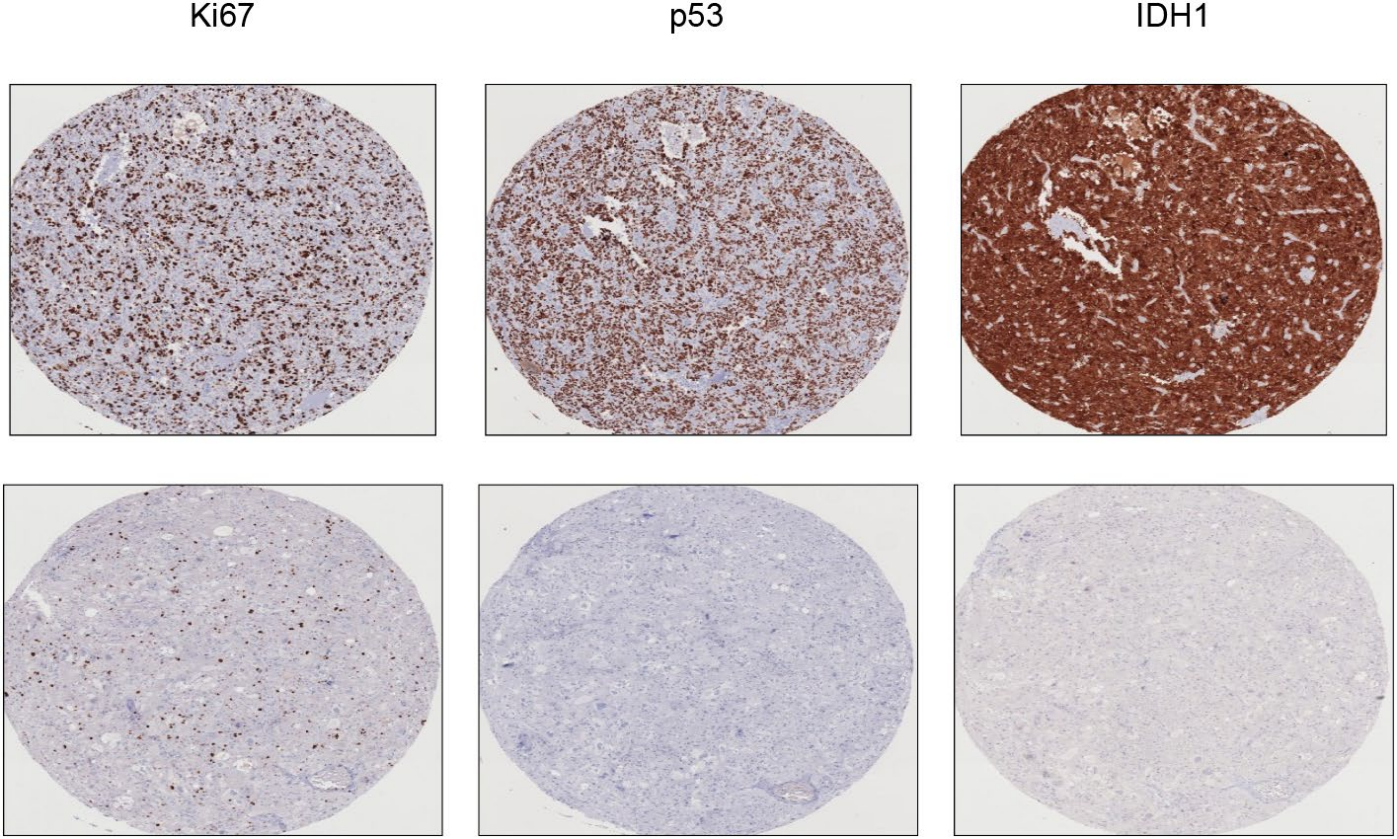
Representative images of Immunohistochemistry for molecular markers of glioblastoma. Images for Ki67 (high above and low below), p53 (positive staining above and negative below), and IDH1 (positive staining above and negative below) are presented.

Finally, we analysed whether MGMT methylation status correlated with different molecular markers such as Ki67 and the mutational status of IDH, p53 y ATRX (Fig. 4). Of those 107 patients who had characterization of MGMT methylation done, we performed immunohistochemistry of Ki67 in 93 (80.9%), p53 in 45 (39.1%), IDH-1 in 35 (30.4%), and ATRX in 17 (14.8%) respectively. None of those variables showed a significant association with MGMT methylation status as it describes in Table 6.

**Table 6:** Association between *O*-6-methylguanine-DNA methyltransferase methylation status and different molecular variables.

	MGMT		p
	Methylated, n (%)	Unmethylated, n (%)	
**KI67, positive cells**			
< 10	11 (55.0)	9 (45.0)	
10–25	23 (45.1)	28 (54.9)	**0.548**
≥ 25	12 (54.5)	10 (45.5)	
**IDH mutation**			
No	13 (41.9)	18 (58.1)	0.441
Yes	2 (50.0)	2 (50.0)	
**P53 mutation**			
No	11 (52.4)	10 (47.6)	
Yes or diffuse	4 (33.5)	8 (66.7)	0.231
Focal or partial	9 (75.0)	3 (25)	
**ATRX mutation**			
No	6 (37.5)	10 (62.5)	
Yes	0 (0.0)	1 (100.0)	0.267

## Discussion

Our results in terms of PFS and OS are in line with the daily reality in clinical practice. Receiving postoperative treatment had a positive impact on survival in our cohort and prognosis was better among patients treated with TMZ for longer exposure. This is in agreement with previous studies^16,17^. Patients who underwent total or subtotal resection lived longer, also in line with previously reported^18,19^. The survival was significantly shorter the worse patients’ general health status at diagnosis. Nonetheless, age was not a prognostic factor in the multivariate analysis; a potential explanation for this is that patients ≥ 65 years of age who received complete treatment, once treated, are in a similar condition to other patients.

In our cohort, MGMT methylation was not found to be a prognostic factor or predictive of TMZ response, unlike what has been described in the major reviews and studies with larger series of patients^6–9^. To our knowledge, this is the first study to date in which MGMT methylation has been found not to be predictive of TMZ response. There are various possible reasons for this finding. The methylation status of the MGMT promoter was not determined in the whole cohort, although in a representative number (107 patients). The methylation sites that affect MGMT expression are not uniformly distributed across CpG islands. In this regard, promoter methylation status may depend on the site at which the surgical sample is taken and if this is small (e.g., a biopsy), the probability of a false negative is greater^20^. The methylation was detected in 48.5% cases slightly higher than that published in larger series^6,8–10,21^. On the other hand, MGMT methylation status has not been assessed homogeneously in all samples and this may have been a limitation. The majority of samples were analysed using methylation-specific PCR (92 out of 107), and the rest have been assessed by pyrosequencing (15 samples). Pyrosequencing has the disadvantage that the appropriate cut-off for classifying a sample as methylated remains unclear^22^. In our hospital, we established three subgroups following a previously described classification^15^. Other research groups have established other cut-offs and have found a correlation between methylation level and response^23–25^. For the same reason, we are also not able to confirm whether the sensitivity of the technique is different and this might have influenced the results. On the other hand, as well as the uncertainly concerning the importance of gene methylation level, it is not known how many and which CpG sites need be methylated to silence gene transcription. Although other studies^23,25^ have found a greater variability in methylation profile, they have not observed correlations between any given methylated CpG site and recurrence or death.

We found that MGMT methylation level increases with age. This finding is in agreement with other studies^23^. Analysing OS, age and methylation status, we found a tendency towards better survival among methylated patients in all age groups. The difference was proportionally larger in patients ≥ 65 years old (11 m vs 6 m), but none of the differences reached significance. Various studies have assessed the correlation between MGMT methylation and survival in patients over 65 years of age^8–10,23^. In all cases, among those treated with TMZ, methylated patients lived significantly longer than unmethylated patients. Most of these studies reported methylation levels in this age group lower than those found in our study (35–46% vs 65.5%)^8–10^ and this might have influenced the final results.

The patients with GBM who benefit most from TMZ are those who do not have MGMT^26^. Nonetheless, in our cohort, MGMT promoter methylation did not affect TMZ-mediated survival. This calls for the consideration of other possibilities, such as the potential test and use of an additional differentially-methylated signature, which has been recently established with prognostic value^27^ or the impact of genetics. For example there is a subtype of GBM that have the CpG island methylation phenotype (G-CIMP phenotype), which is associated with GBM with IDH mutation and hypermethylation of MGMT^16^. Similarly, epigenetic inactivation of MGMT can induce point mutations in TP53. Additionally, an study associates the deletion of an enhancer that is located between the promoters of the Ki67 and MGMT index with reduced levels of expression of MGMT and lower expression of Ki67^28^. In our study, we did not detect association between the methylation status of MGMT promoter and the expression or mutation of Ki67, p53, IDH1 and ATRX molecular factors, which are relevant biological markers in glioblastoma. However, except for Ki67 the rest of the determinations have been performed in relative short number and this fact makes the sample not significant enough to draw conclusions regarding the link between MGMT and genetics. One hypothesis would be that the activity of MGMT promoter methylation is cancelled out by other yet unidentified effects. For example, it is known that epigenetic silencing of MGMT may have tumour-inducing effect^19^, and this could explain the lack of differences in our cohort. Another potential explanation for our results is that the regulation of MGMT methylation differs in our cohort. In the study of Hegi et al., 13.8% of unmethylated patients treated with TMZ lived at least 2 years^6^. This observation suggests that the methylation test used may be not be sufficiently sensitive to identify all responders and/or that there are mechanisms other than methylation that also suppress MGMT. For example, the transcription and subsequent expression of the MGMT protein may be affected by genetic factors such as single nucleotide polymorphisms (SNPs) in the gene promoter region. Genome-wide association studies have identified some SNPs that increase the risk of developing GBM^26,29^. Among these, the MGMT promoter SNP rs16906252 seems to play a key role in the acquisition of MGMT methylation, and hence, increases the risk of developing MGMT-methylated GBM^29^. As well as SNPs, microRNAs play a role in the post-transcriptional regulation of MGMT^22,30^. It seems that the direct binding of specific microRNA to the 3′ terminal region of MGMT transcripts may reduce mRNA stability. Additionally, expression of MGMT could be altered by other epigenetic mechanisms such as methylation of lysine residues of histones, leading to either transcriptional activation or repression depending on which histone residue is modified. For example, methylation of lysine residue 9 within histone 3 (H3K9) inhibits transcription of MGMT promotor. Methylation of lysine residues is performed by specific histone lysine N-methyltransferases. In this line SUV39H1 and SETDB1, the principal histone lysine N-methyltransferases which take part in the regulation of the trimethylation of H3K9, are upregulated in glioma cells and their suppression reduces tumor cell properties^31^.

In summary, in our cohort of patients, MGMT methylation did not have an impact on PFS or OS. Patients treated with TMZ with MGMT methylation did not have a lower rate of recurrence or better survival than those in whom there was no methylation. Treatment with TMZ and complete resection were positive prognostic factors; on the other hand, survival was significantly shorter in patients with the poorest general status. Analysing time to recurrence, under-65-year-olds treated with TMZ had later recurrence, while complete resection was a predictive factor in patients ≥ 65 years of age.

## Supplementary information

Supplementary Figure 1.

## Data Availability

The data that support the findings of this study are available from the corresponding author upon reasonable request.
