# Supplementary material for: Methylation of MGMT promoter does not predict response to temozolomide in patients with glioblastoma in Donostia Hospital
Source: Sci Rep. 2020 Oct 28;10:18445. doi: 10.1038/s41598-020-75477-9 (PMC7595088; doi:10.1038/s41598-020-75477-9)
Supplement: Supplementary file 1 — Supplementary Figure 1. [file 41598_2020_75477_MOESM1_ESM.pptx]

## Slide 1
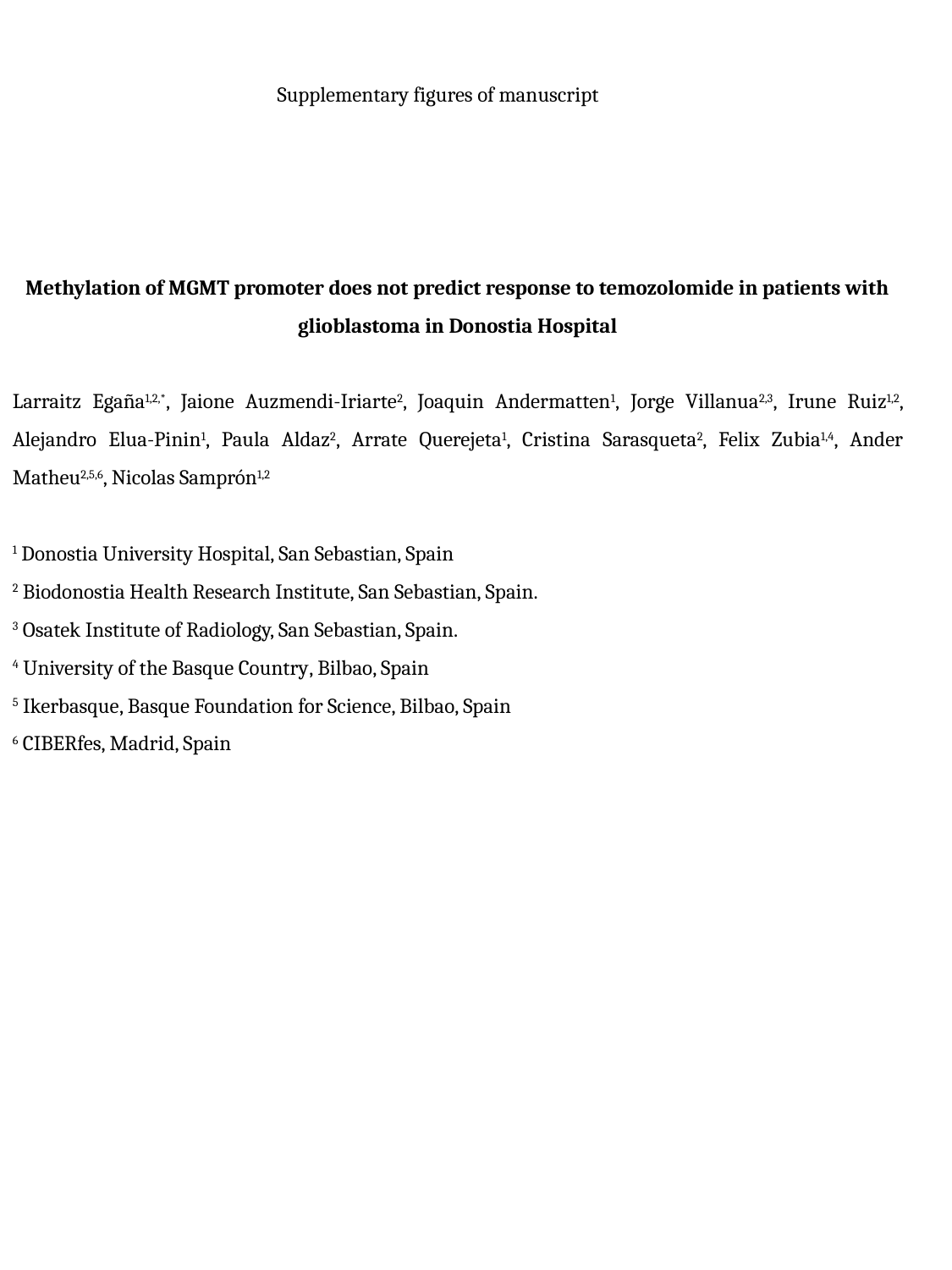

Supplementary figures of manuscript
Methylation of MGMT promoter does not predict response to temozolomide in patients with glioblastoma in Donostia Hospital
Larraitz Egaña1,2,*, Jaione Auzmendi-Iriarte2, Joaquin Andermatten1, Jorge Villanua2,3, Irune Ruiz1,2, Alejandro Elua-Pinin1, Paula Aldaz2, Arrate Querejeta1, Cristina Sarasqueta2, Felix Zubia1,4, Ander Matheu2,5,6, Nicolas Samprón1,2
1 Donostia University Hospital, San Sebastian, Spain
2 Biodonostia Health Research Institute, San Sebastian, Spain.
3 Osatek Institute of Radiology, San Sebastian, Spain.
4 University of the Basque Country, Bilbao, Spain
5 Ikerbasque, Basque Foundation for Science, Bilbao, Spain
6 CIBERfes, Madrid, Spain

## Slide 2
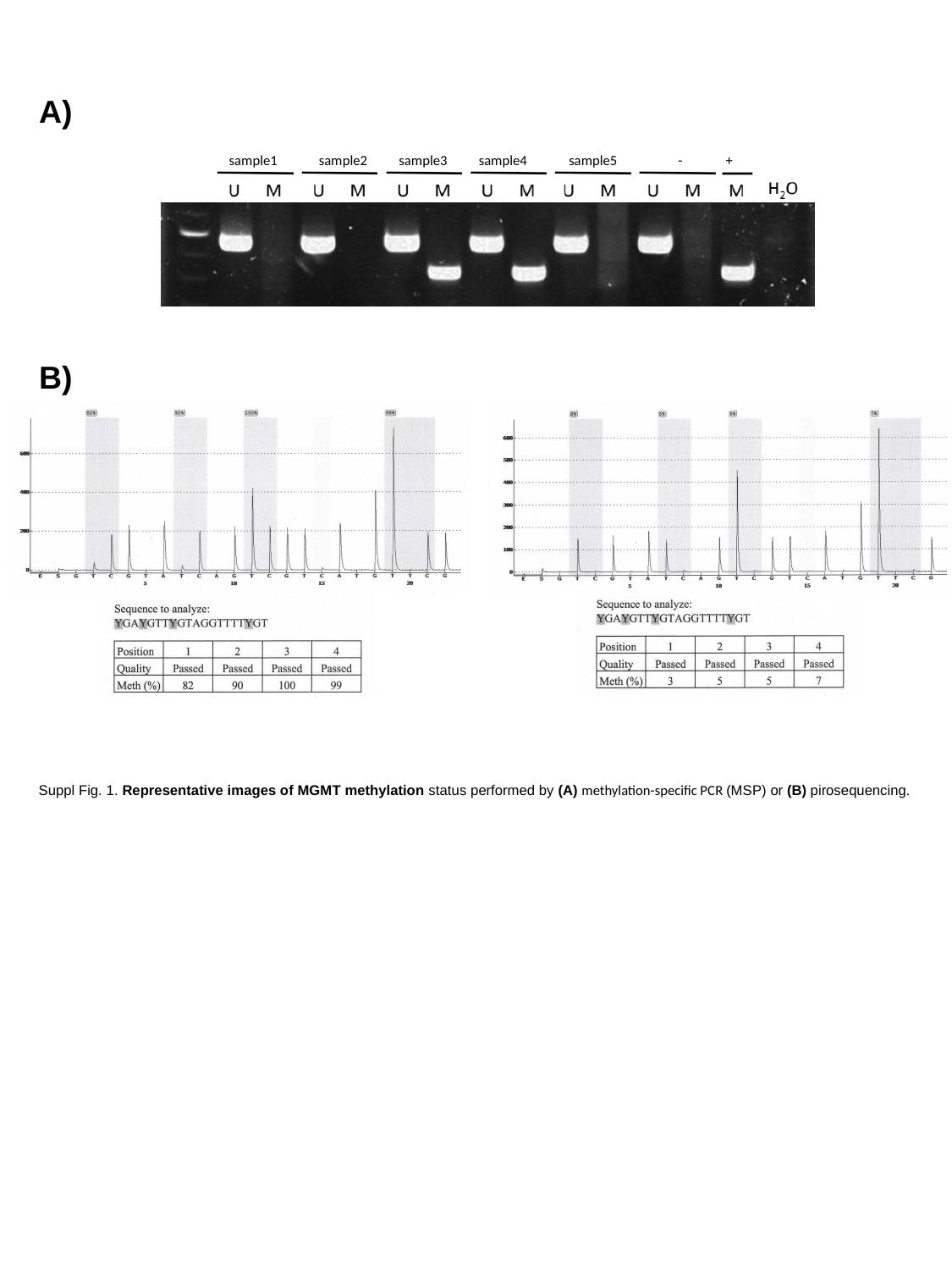

A)
sample1
sample2
sample3
sample4
sample5
-
+
B)
Suppl Fig. 1. Representative images of MGMT methylation status performed by (A) methylation-specific PCR (MSP) or (B) pirosequencing.
